# Gender May Influence the Immunosuppressive Actions of Prednisone in Young Patients With Inflammatory Bowel Disease

**DOI:** 10.3389/fimmu.2021.673068

**Published:** 2021-05-13

**Authors:** Marianna Lucafò, Matteo Bramuzzo, Davide Selvestrel, Prisca Da Lozzo, Giuliana Decorti, Gabriele Stocco

**Affiliations:** ^1^ Institute for Maternal and Child Health - IRCCS Burlo Garofolo, Trieste, Italy; ^2^ Department of Life Sciences, University of Trieste, Trieste, Italy; ^3^ Department of Medical, Surgical and Health Sciences, University of Trieste, Trieste, Italy

**Keywords:** glucocorticoids, inflammatory bowel disease, gender, age, GILZ, prednisone

## Abstract

Although the use of glucocorticoids (GC) is well established, the therapeutic response to these agents often shows important interindividual differences, in particular among young patients with inflammatory bowel diseases (IBD). Currently, GC resistance or dependence cannot be predicted by clinical or laboratory findings. The aim of this study was to investigate the association of gender and age with GC efficacy and with the expression of Glucocorticoid-Induced Leucine Zipper (GILZ). One hundred thirty patients (mean age at enrolment 12.6 years, 53 Crohn’s disease, 70 males) were enrolled in this retrospective study. IBD patients with active disease despite prednisone at a daily dose of up to 2 mg/kg over a period of 4 weeks were defined as steroid resistant. Patients who initially responded but relapsed upon dose reduction were considered steroid-dependent. Total RNA was extracted from biopsies of 14 patients (9 males) and the levels of GILZ mRNA were evaluated by real-time PCR. Association between clinical response to prednisone and the considered demographic variables was evaluated using logistic regression models. After 4 weeks of treatment, 112 patients were responders to prednisone and 18 were resistant; at this time-point, resistant patients were older than responders (p=0.032). After 12 weeks, 42, 71 and 12 patients were sensitive, dependent and resistant respectively; at this time-point, females were more prone than males to develop prednisone dependence vs a good response (p=0.028) while age had no effect. Age was associated with response both at 4 and 12 weeks in the subgroups of females: resistant patients were older than sensitive ones at 4 weeks (p=0.02). Likewise, at 12 weeks of therapy, dependent patients resulted older than sensitive ones (p=0.05). No association of age with prednisone response was found in males. In a subgroup of 14 patients (5 females), GILZ mRNA expression in intestinal biopsies was higher in males (p=0.0031). Patients with unfavorable response (7) presented lower GILZ expression at disease onset in comparison to the responder group (p=0.017). Older females with IBD have a higher incidence of prednisone unfavorable response and reduced intestinal expression of the GC pharmacodynamic marker GILZ.

## Introduction

Inflammatory bowel disease (IBD) is a chronic idiopathic inflammation of the intestinal tract, characterized by relapses and remissions, that comprises two disorders, Crohn’s disease (CD) and ulcerative colitis (UC). The disease has a peak onset in subjects 15 to 30 years old, and its incidence is rising in the young population with approximately 25% of patients with IBD presenting before the age of 20 (1). Patients with pediatric-onset IBD tend to present with more extensive anatomic involvement, more active disease course and often require a more aggressive treatment compared to patients with adult onset disease (2). The etiology of CD and UC is often undefined and different genetic and environmental causes may result in a similar clinical outcome (3). At present, a curative therapy for IBD does not exist and the treatment aims to induce and maintain the disease in an inactive state. For this purpose, many immunosuppressants are currently employed due to the clear contribution of an uncontrolled immune response in the pathogenesis of both CD and UC (4). Despite the introduction in clinical practice of highly effective biological drugs, such as anti-TNFα agents, glucocorticoids (GCs) are still used to induce disease remission in young patients with moderate to severe active CD and UC (5, 6). GCs are steroid hormones that passively diffuse across plasma membranes and act by binding the GC receptor (GR) (7) which becomes able to translocate into the nucleus and binds as homodimer to DNA, in correspondence of GC-responsive elements (GREs), localized in the promoter region of target genes (8, 9). This binding activates the transcription of anti-inflammatory proteins such as glucocorticoid-induced leucine zipper (GILZ), interleukin-10 (IL-10), annexin 1 and enzymes involved in gluconeogenesis (10, 11), and reduces the expression of pro-inflammatory cytokines (IL-1, IL-2, IL-6, TNFα) and inflammatory prostaglandins (12).

Although the use of GCs is well established in many diseases due to their efficacy, the therapeutic response to these agents often shows important interindividual differences between patients (13–15), especially in pediatric inflammatory diseases (6). According to the clinical response, young IBD patients treated with GCs can be classified as steroid sensitive (SS) or steroid resistant (SR) within the first 30 days of treatment (16). A high percentage of patients who initially respond to GC therapy is unable to reduce steroids within 3 months of starting due to frequent relapses: these patients are classified as steroid-dependent (SD) (13, 17).

Steroid resistance is common in young patients with IBD, with an incidence of 6-20% of patients, however, a specific predictor of GC response has not yet been identified (18, 19). While resistance to GC has been widely studied both in pediatric and adult patients, few data are available for steroid dependency (17). Demographic characteristics, in particular age and gender, have been associated with GC levels (20), response (18) and side effects (21) but very few data are available on the young population affected by IBD. Moreover, the molecular mechanisms of the interindividual variability in GC response is still poorly understood in these patients (22). Polymorphisms in genes involved in the GC pathway together with the expression of candidate mRNAs and epigenetic factors have been previously reported as possible mechanisms involved in steroid resistance even though none of them had entered in the clinical practice as biomarker of response (18, 22–24).

Therefore, the aim of this study was to evaluate the association of age and gender with prednisone efficacy in a large cohort of children with IBD and to investigate gender-specific differences in GR–mediated gene expression in these patients, considering in particular GILZ expression in intestinal biopsies.

## Materials and Methods

### Patients Characteristics

130 young patients with IBD were enrolled by the Gastroenterology Unit of the Pediatric Hospital “Burlo Garofolo” in Trieste, Italy between July 2001 and February 2019. The study was conducted in accordance with the Declaration of Helsinki, and the protocol was approved by the Institutional Ethics Committee. All subjects and/or parents gave their informed consent for inclusion before they participated in the study. The inclusion criteria were age less than 25 years, previous diagnosis of IBD and initial treatment with prednisone for 4 weeks at the standard dose (1 to 2 mg/kg/day) and 8 to 11 weeks of tapering. Steroids were administered as induction treatment in patients with ileo-colic or colonic CD when exclusive enteral nutrition was not an option and in patients with moderate or severe UC according to the pediatric ulcerative colitis activity index (PUCAI) and the pediatric Crohn’s disease activity index (PCDAI) or to the physician global assessment.

Clinical response to prednisone was assessed after 4 and 12 weeks of therapy. Patients were considered responsive to treatment when a drop of at least 50% from the basal clinical score was observed. In case of clinical score unavailability, the physician global assessment definitions of “remission” and “response” or “persistent active disease” were used. Patients who initially responded to prednisone therapy but were unable to reduce steroids within 3 months of starting treatment were classified as steroid-dependent. Patients not classified as responders or dependent to prednisone, were considered resistant.

### Total RNA Isolation

Total RNA was extracted from intestinal biopsies of 14 patients using TRIzol reagent (Thermo Scientific, Carlsbad, CA, USA) according to manufacturer’s instructions. The RNA concentration and purity were calculated by Nano Drop instrument (NanoDrop 2000, EuroClone, Milan, Italy).

### Quantitative Real-Time PCR

Expression levels of GILZ was evaluated by real-time RT-PCR TaqMan^®^ analysis using the CFX96 real-time system-C1000 Thermal Cycler (Bio-Rad Laboratories, Hercules, CA, USA). The reverse transcription reaction was carried out with the High Capacity RNA-to-cDNA Kit (Applied Biosystem, Foster City, CA, USA) and the real-time PCR was performed in triplicate using the TaqMan^®^ Gene Expression Assay to assess GILZ mRNA expressions, according to the manufacturer’s instructions. The thermal cycling conditions for TaqMan assays were as follows: 2 min at 50°C and 10 min at 95°C followed by 40 cycles at 95°C for 15 s and 60°C for 60 s. The expression levels of GILZ was evaluated using the comparative Ct method (2−ΔCt method) and normalized using the RPLP0 gene.

### Statistical Analysis

Statistical analysis was performed using the software R, version 3.6.1.

Analysis for association of clinical response, evaluated as a categorical variable, was performed by logistic regression. For these analyses, binomial models were generated using response to prednisone as the dependent variable and the clinical or demographic covariate of interest (i.e., gender and type of IBD) as the independent variable. Odds ratios were calculated from estimates of the logistic regression models.

For the analysis considering continuous variables (i.e. age and GILZ expression) linear models of the Gaussian family were used. Before applying linear models, normality of the continuous variable was assessed. All p-values < 0.05 were considered statistically significant.

## Results

### Patients Enrolled

One hundred thirty IBD patients treated with prednisone were enrolled in this retrospective study; baseline characteristics of the patients are shown in **Table 1**.

**Table 1:** Demographic and clinical characteristics of the patients enrolled in the study.

		All patients (n = 130)
Age (mean, range)		12.6, 1.2-22.2
Gender	Female (%)	60 (46.2%)
	Male (%)	70 (53.8%)
Type of IBD	Crohn’s disease (%)	53 (40.8%)
	Ulcerative colitis (%)	77 (59.2%)
Clinical Score ^(data available for 94 patients)^	PUCAI (mean, range)	41.4, 0-85
	PCDAI (mean, range)	34.0, 2.5-65

### Response to Prednisone: Association With Clinical and Demographic Data

The clinical response was evaluated after treatment with prednisone 1–2 mg/kg/day and after 4 (initial response) and 12 weeks of therapy (tapering dose).

Among 130 patients enrolled, after 4 weeks of treatment, 112 (86.2%) were responder to prednisone and 18 (13.9%) were resistant. Out of the 130 patients age was available only for 93 sensitive and 17 resistant patients for a total of 110 subjects.

**Figure 1:**
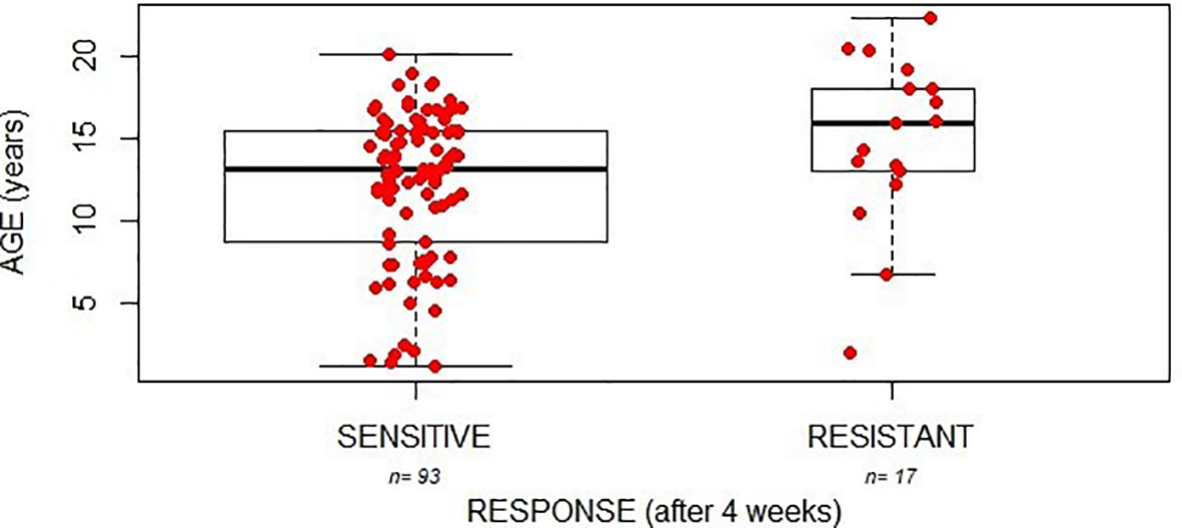
Initial response to glucocorticoids (4 weeks of therapy) and age of patients.

Interestingly, a significant association was found between the clinical response after 4 weeks of treatment and age: in particular, resistant patients resulted to be older than sensitive ones (mean age: 14.9 and 12.1 respectively, linear model p=0.032; **Figure 1**). No significant association was highlighted with sex, disease severity or type of IBD and clinical response at 4 weeks of treatment.

After tapering (12 weeks), 42 (32.3%), 71 (54.6%) and 12 (9.2%) patients were sensitive, dependent and resistant respectively. For 5 (3.8%) patients the information related to the clinical response at 12 weeks was not available for lack of scheduled visit.

Unlike what was observed with the response at 4 weeks of treatment, no significant association was found between clinical response after 12 weeks of treatment and age. A significant association was on the contrary detected between the clinical response at 12 weeks of treatment and the type of IBD; in particular, patients with UC were more GC dependent in comparison to patients with CD (logistic regression p=0.018 OR: 2.62; CI: 1.19 - 5.78). On the other hand, no significant difference was observed in the incidence of resistance according to age, disease severity or IBD type.

Among the 130 patients, 70 (53.9%) were concomitantly treated with prednisone and other medications and in particular: 55 with azathioprine, 6 with thalidomide, 3 with infliximab, 1 with adalimumab, 1 with methotrexate and 4 with topical therapy (budesonide). No association was found between the combination therapy with other immunomodulators and the response to prednisone.

Interestingly, females were more prone than males to develop prednisone dependence after 12 weeks of treatment (logistic regression p=0.028; OR: 2.44; CI: 1.10 - 5.26). Incidence of resistance was not affected by gender.

Patients were further stratified into pre-pubescent (≤ 13 years) and post-pubescent (> 13 years) categories for all analyses, but no statistical association was found, probably due to resulting sample size of the subgroups.

Multivariate analysis shows that sex (logistic regression, sensitive *vs* dependent p=0.03; OR: 2.38; CI: 1.06 - 5.26) and the type of IBD (logistic regression, sensitive *vs* dependent p=0.02; OR: 2.59; CI: 1.16 – 5.80) are independently associated with the clinical response at 12 weeks (**Table 2**).

**Table 2:** Multivariate analysis considering all covariates significant in the univariate analysis.

Covariate	Comparison	p-value (OR; CI)
Sex	Sens vs Dep	0.03 (2.38; 1.06 - 5.26)
(F vs M)	Sens vs Res	0.30 (2.00; 0.54 – 7.14)
Type of IBD	Sens vs Dep	0.02 (2.59; 1.16 – 5.80)
(UC vs CD)	Sens vs Res	0.53 (1.53; 0.41 – 5.62)

**Figure 2:**
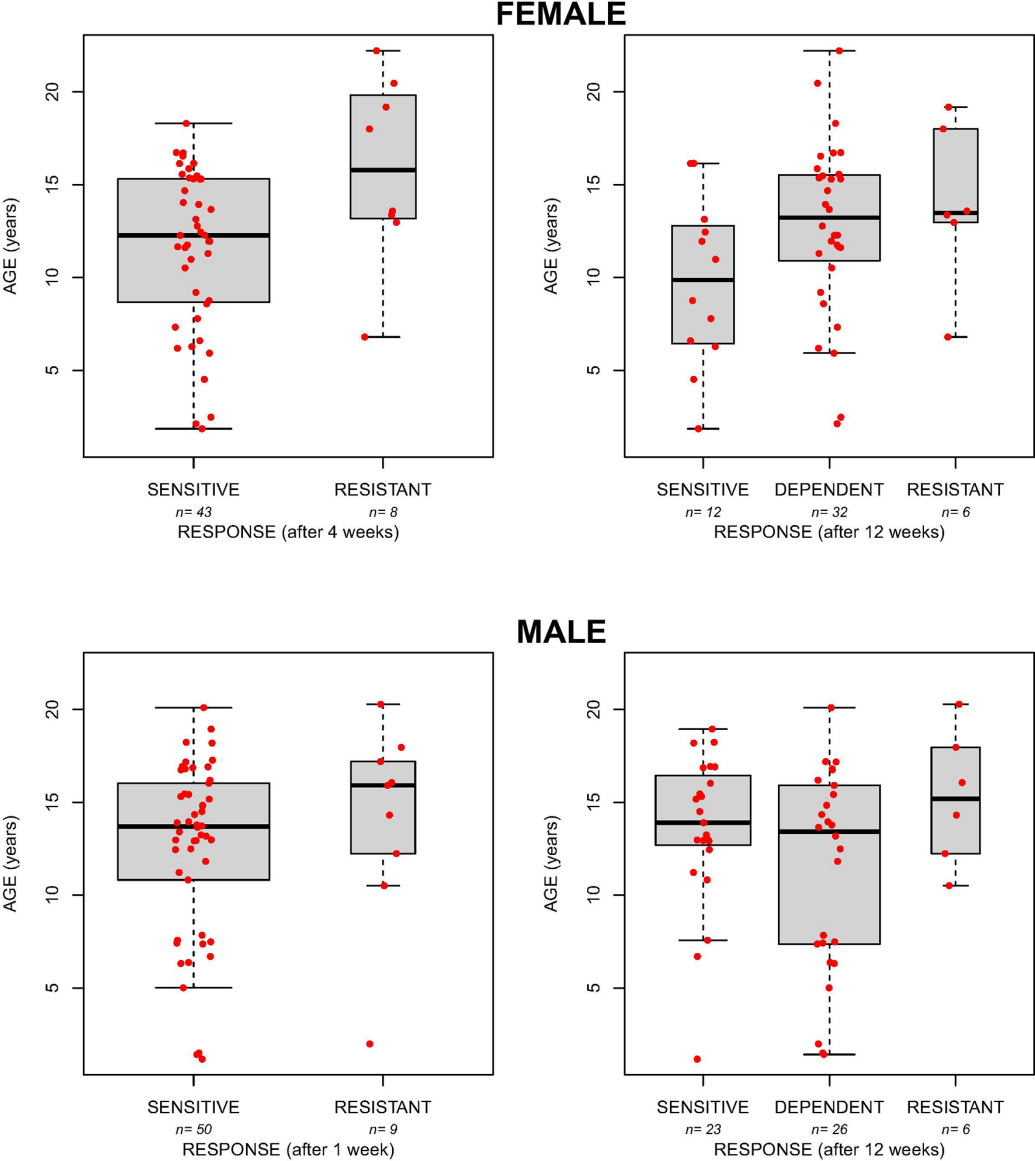
The boxplots show the age in female and male patients sensitive, resistant or dependent to GC after 4 and 12 weeks of therapy.

Given the association between patient age and GC response (after 4 weeks of treatment, **Figure 1**) and the association with gender considering the clinical response after 12 weeks of treatment, we evaluated a potential effect of age on the response to prednisone in the subgroups of female and male patients. As reported in **Figure 2**, age is associated with response at 4 and 12 weeks in females. In particular, resistant patients resulted to be older than sensitive ones at 4 weeks of treatment (mean age: 11.6 and 15.8 respectively; linear model p=0.02). Likewise, at 12 weeks of therapy dependent patients resulted to be older than sensitive ones (mean age: 15.5 and 12.6 respectively, linear model p=0.05; **Figure 2**) and a similar trend was observed also for resistant patients in comparison with the responder group (mean age 16.9; linear model p=0.07; **Figure 2**).

No association was found between age and the response to prednisone at both time points in the group of males (**Figure 2**).

### Gender-Specific Mechanism Controlling GC Receptor–Mediated GILZ Expression

**Figure 3:**
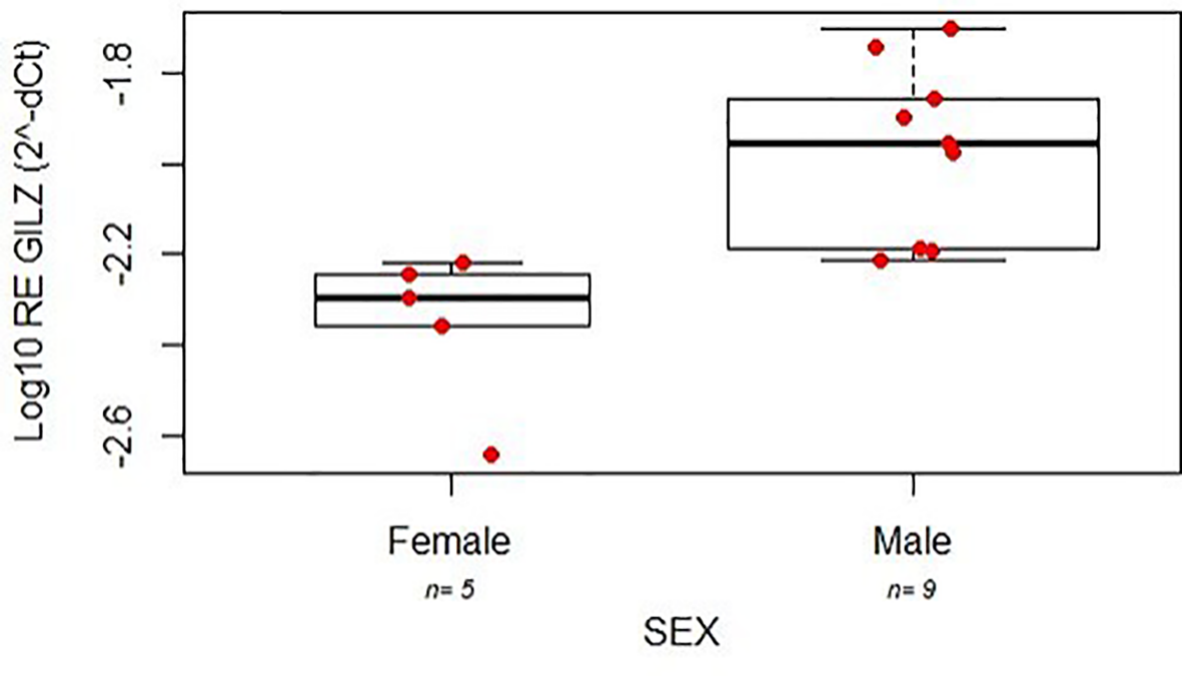
Expression (relative log expression) of GILZ mRNA between the two genders affected by IBD.

To investigate on the gender-specific actions of prednisone in IBD patients, we evaluated in intestinal biopsies at disease onset the levels of the GC target gene GILZ in a subgroup of 14 patients (5 females, 3 CD; **Supplementary Table 1**), belonging to the same cohort of patients described above; no significant difference was observed in the demographic characteristics between the whole cohort and this subgroup. As reported in **Figure 3**, GILZ mRNA expression was lower in females in comparison to males (linear model p=0.0031), highlighting a different activity of the GC receptor between genders.

**Figure 4:**
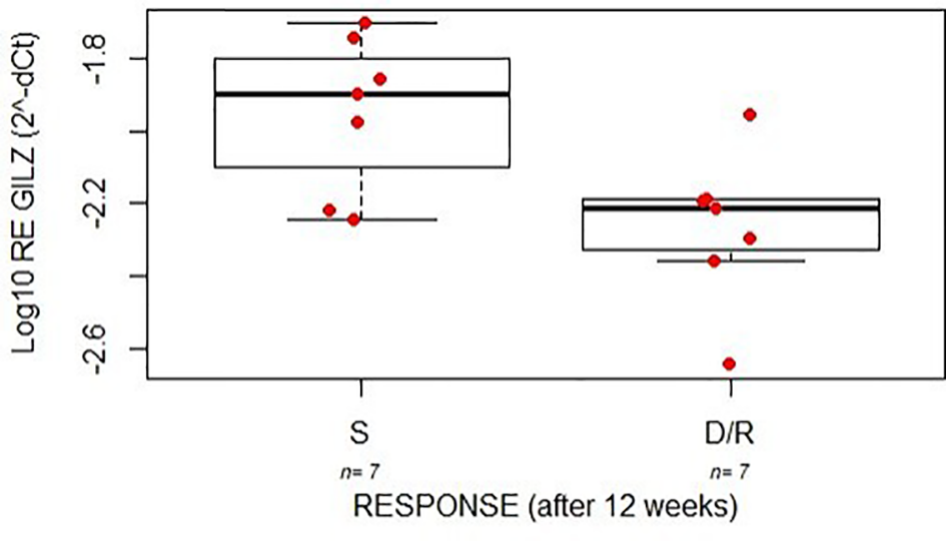
The boxplots indicate the expression level (relative log expression) of GILZ between good (S) and poor (D/R) responders after 12 weeks of treatment with GC in a cohort of young patients with IBD.

Interestingly, patients with unfavorable response (dependent + resistant) presented lower GILZ levels at disease onset in comparison to the responder group (linear model p=0.017; **Figure 4**).

**Figure 5:**
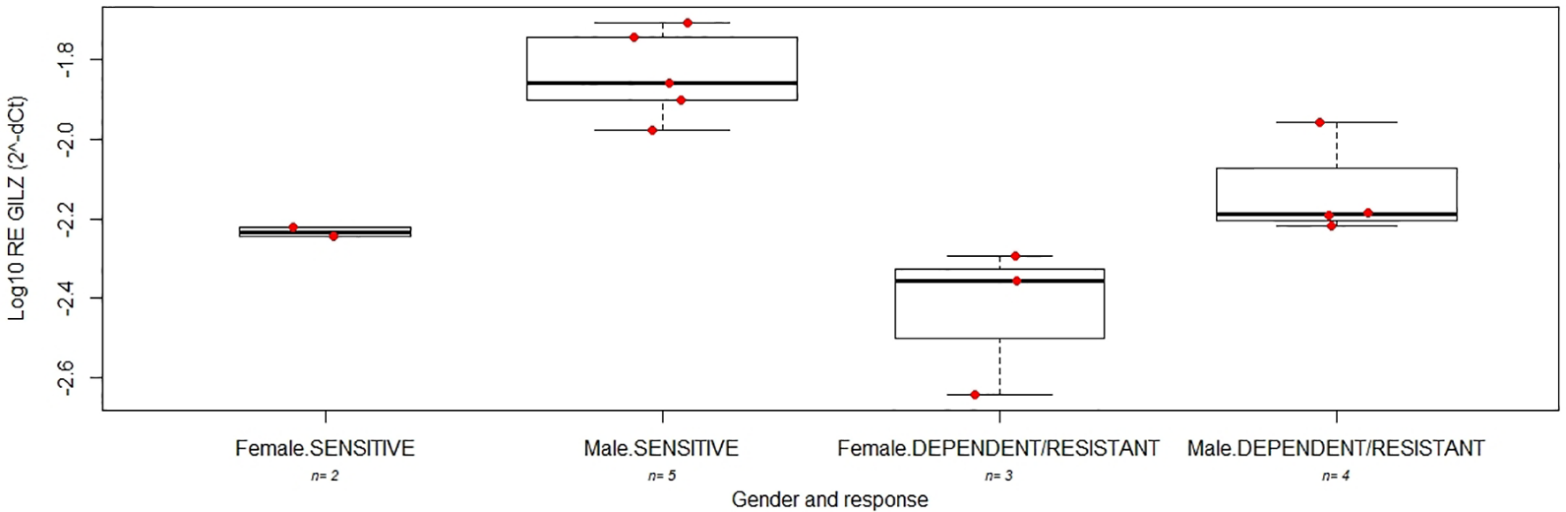
The boxplots indicate the expression level of GILZ between good (SENSITIVE) and poor (DEPENDENT/RESISTANT) responders after 12 weeks of treatment with GC in female and male young patients with IBD.

Association of GILZ levels with gender and response to GCs was confirmed also by multivariate analysis (adjusted p-value respectively 0.00053 and 0.0024; **Figure 5**).

## Discussion

The use of GCs in young patients with IBD is mostly extrapolated from the experience in adults although many differences have been described and children are more sensitive to GC therapy-related side effects than adults (25). Despite the numerous studies conducted to investigate the underlying mechanism of resistance to GCs, no demographic, clinical or molecular biomarker is available yet to predict response or the risk of developing adverse events (4, 16). Moreover, while the molecular basis of GC resistance have been explored, the molecular mechanisms behind GC dependence are poorly investigated (5) and only few studies have been conducted in pediatric patients with IBD (17).

In our study, performed in a cohort of young patients, age and gender seem to be important contributing factors to prednisone response. In particular, resistant patients resulted to be older than sensitive ones after the initial 4 weeks of prednisone treatment, while females were more prone than males to develop dependence after tapering. A significant association was also reported between the type of IBD and clinical response at 12 weeks of treatment, but the multivariate analysis showed an independent effect of gender and the type of IBD.

The levels of endogenous GCs have been associated with age and gender, suggesting their important role in regulating GC secretion (26). Cortisol levels increase progressively with age in particular in women (20). However, the influence of age and sex on GC pharmacokinetic and pharmacodynamic properties have been reported only in adult subjects though these evidences do not suggest the need for dosage adjustments. Nowadays, the only data regarding age and gender differences in GC response in young patients with IBD were previously published by our group (18). In particular, it was reported that in IBD male patients with an age at disease onset above 7.5 years, response to GC therapy was significantly more frequent than females.

In the present study an association of age with the response to prednisone in females was highlighted: female responder patients after both 4 and 12 weeks of treatment, were significantly younger, while this effect of age was not observed in males.

The influence of female hormonal status in IBD activity is recognized, but no studies have investigated how sex hormones modulate therapeutic response (27–30). Of interest, Altemus et al. observed a decrease in GR mRNA in the luteal phase, characterized by an increase of progesterone and, to a lesser extent, of estrogen levels (31). Moreover, the female sex steroid hormone progesterone has been shown to antagonize GC effects increasing the dissociation rate of GC from the GR and inhibiting its translocation into the nucleus (32, 33). Considering the crucial role of these hormones, GCs may not work properly in adolescent girls and young women with IBD.

To investigate on the gender-specific actions of prednisone in IBD patients, we analyzed the expression of the mRNA of the anti-inflammatory GC-induced gene GILZ in intestinal biopsies obtained before starting GC treatment of a subgroup of female and male patients of the same cohort, as a predictive pharmacodynamic marker of GC efficacy. GILZ levels were higher in males in comparison to females highlighting a different activity of the GC receptor between genders.

Sexually dimorphic GR signaling was already studied by pioneering investigations of the Cidlowski group (34–36). Results from their study indicate that estradiol antagonizes induction of GILZ gene expression providing evidence of its role also in the immune modulating functions of GCs (36).

Moreover, Cidlowski and collaborators demonstrated that ovarian hormones can influence the anti-inflammatory actions of GCs in the context of a rat adrenalectomized model of sepsis and that the anti-inflammatory actions of GCs are more effective in males (34). In particular, specific profiles of GC-regulated gene expression have been observed for females and males confirming a different gender-specific mechanism controlling GR–mediated gene expression. Even if the mechanisms that drive gender-specific effects of GC are still unclear, Cidlowski and collaborators showed a higher nuclear translocation of the GR in males after administration of the proinflammatory stimulus lipopolysaccharide compared to female mice, suggesting that the activation of GR is sex-specific.

The importance of the transcriptional activity of the GR in GC effectiveness is confirmed by the fact that in our study patients with unfavorable response presented lower GILZ levels at disease onset in comparison to the responder group. GILZ is expressed in all peripheral blood cells and in several other non-lymphoid tissues including cell types present in the biopsies such as immune cells (T-lymphocytes and monocytes) and intestinal cells (37). This is the first study to consider the association of GILZ expression on GC sensitivity in colon biopsies from a cohort of pediatric patients with IBD and no data about the modulation of GILZ levels during GC treatment in IBD patients have been published yet. In other diseases, in particular systemic lupus erythematosus and alcohol‐induced liver disease, GILZ was overexpressed in blood cells of patients under treatment with GC, but no association with the clinical response have been analyzed (38, 39).

The role of GILZ has been studied in several mouse models of IBD using dinitrobenzene sulfonic acid-induced colitis (40). In particular, the severity of colonic inflammation was diminished in transgenic mice overexpressing GILZ and exacerbated in GILZ-conditional knock-out mice with a T-cell specific GILZ deletion highlighting its anti-inflammatory effects that mimic those of GCs (41, 42). An opposite effect of GILZ was observed in oxazolone-induced colitis which is a Th2-dependent model of chronic inflammation where more severe disease was induced in mice overexpressing GILZ compared to the wild type mice suggesting that GILZ could have a protective role in Th1-mediated models of colitis (41). Further validation studies will help to explore the promising role of GILZ as marker of GC response in IBD patients as already proposed for other pathologies (43, 44) evaluating its levels also in the blood cells to avoid an invasive examination.

Our study has some limitations starting from its retrospective design. Unfortunately, no data about specific treatment administered before prednisone are available. Clinical score was not available for 36 of 130 patients before the treatment with prednisone because laboratory data from the retrospective analysis were incomplete. Moreover, the association of GILZ expression on GC sensitivity was limited to a small sample size and mechanistic data to confirm the role of GILZ in GC response are needed.

In the present study we have demonstrated that age and gender are important variables affecting response to prednisone in young patients with IBD even though further investigation on the impact of sex hormones on therapeutic outcomes and disease course are needed. Our results support the hypothesis that the anti-inflammatory actions of GCs are reduced in female adolescents probably due to a lower activity of the GR. Furthermore, sex hormones, in particular estrogen and progesterone, and their fluctuation with age and critical phases such as puberty, might contribute to the differential sensitivity to GCs. The study is descriptive and data interpretation including links with female hormones are speculative, more in-depth investigation are therefore needed to establish the exact molecular mechanisms of these difference.

## Data Availability Statement

The raw data supporting the conclusions of this article will be made available by the authors, without undue reservation.

## Ethics Statement

The studies involving human participants were reviewed and approved by Local ethical committee (Comitato indipendente per la bioetica, Istituto di Ricovero e Cura a Carattere Scientifico materno infantile Burlo Garofolo, Trieste, Italy). Approval for the study (Prot 2198; approval date: 17 September 2013) was provided. All patients participated in this study in accordance with the principles outlined in the Declaration of Helsinki, and written informed consent was obtained from each participating patient and/or their parents or guardians. Written informed consent to participate in this study was provided by the participants’ legal guardian/next of kin.

## Author Contributions

ML and GS: study conception and design. ML, DS, and GS: acquisition, analysis, and interpretation of the data, and drafted the initial manuscript. ML, MB, and PD: acquisition of the data. GD and GS: critical discussion and study supervision. All authors contributed to the article and approved the submitted version.

## Funding

This work was supported by the Institute for Maternal and Child Health “Burlo Garofolo,” Trieste, Italy (grant number RC 10/19).

## Conflict of Interest

The authors declare that the research was conducted in the absence of any commercial or financial relationships that could be construed as a potential conflict of interest.
