# Supplementary material for: Gender May Influence the Immunosuppressive Actions of Prednisone in Young Patients With Inflammatory Bowel Disease
Source: Front Immunol. 2021 May 13;12:673068. doi: 10.3389/fimmu.2021.673068 (PMC8158435; doi:10.3389/fimmu.2021.673068)
Supplement: Supplementary file 1 [file Table_1.docx]

**Supplementary Table 1**. Demographic and clinical characteristics of the patient subgroups.

|  | | All patients (n = 14) |
| --- | --- | --- |
| Age (mean, range) | | 14.6, 6.3-18.9 |
| Gender | Female (%) | 5 (35.7%) |
|  | Male (%) | 9 (64.3%) |
| Type of IBD | Crohn’s disease (%) | 3 (21.4%) |
|  | Ulcerative colitis (%) | 11 (78.6%) |
